# Nonconformity of biofilm formation in vivo and in vitro based on *Staphylococcus aureus* accessory gene regulator status

**DOI:** 10.1038/s41598-022-05382-w

**Published:** 2022-01-24

**Authors:** S. Caroline Jordan, Pamela R. Hall, Seth M. Daly

**Affiliations:** Department of Pharmaceutical Sciences, University of New Mexico College of Pharmacy, Albuquerque, NM 87131 USA

**Keywords:** Biofilms, Pathogens

## Abstract

*Staphylococcus aureus* is an opportunistic, pathogenic bacteria that causes significant morbidity and mortality. As antibiotic resistance by *S. aureus* continues to be a serious concern, developing novel drug therapies to combat these infections is vital. Quorum sensing inhibitors (QSI) dampen *S. aureus* virulence and facilitate clearance by the host immune system by blocking quorum sensing signaling that promotes upregulation of virulence genes controlled by the accessory gene regulator (*agr*) operon. While QSIs have shown therapeutic promise in mouse models of *S. aureus* skin infection, their further development has been hampered by the suggestion that *agr* inhibition promotes biofilm formation. In these studies, we investigated the relationship between *agr* function and biofilm growth across various *S. aureus* strains and experimental conditions, including in a mouse model of implant-associated infection. We found that *agr* deletion was associated with the presence of increased biofilm only under narrow in vitro conditions and, crucially, was not associated with enhanced biofilm development or enhanced morbidity in vivo.

## Introduction

*Staphylococcus* aureus, both methicillin-sensitive (MSSA) and methicillin-resistant (MRSA), is one of the most common causes of healthcare-associated and community-acquired infections and is associated with significant morbidity and mortality^1^. In a 2019 report on antibiotic resistance, the Centers for Disease Control and Prevention designated MRSA a serious public health threat and estimated that it was responsible for more than 300,000 infections in hospitalized patients, $1.7 billion in healthcare costs and more than 10,000 deaths in the United States in 2017^2^. Biofilms formed by *S. aureus*, which are intrinsically recalcitrant to antibiotics, represent a large portion of infections and medical devices, such as catheters and prostheses, being particularly problematic^3–5^. As antibiotic resistance, especially multi-drug resistance, becomes increasingly prevalent, the investigation and development of novel therapies to combat these infections is essential. *S. aureus* infections are no exception and one alternative target is the accessory gene regulator (*agr*) operon which encodes a quorum sensing system that, while not directly affecting survival, regulates the production of many of the virulence factors utilized by *S. aureus* during invasive infections (reviewed in^6^). There are four distinct *agr* types (*agr*1-4) and, importantly, isolates of all four types can cause disease in humans; each allele differs in the secreted hormone and its cognate receptor^6^.

Compounds that inhibit *S. aureus* quorum sensing, and therefore suppress *agr*-dependent virulence factor production, have been proposed as a means of reducing *S. aureus* infection severity and promoting immune system clearance of the pathogen^7–10^. However, development of quorum sensing inhibitors (QSI) as viable therapeutic options has been perhaps unfairly criticized by research suggesting that inhibiting the *agr* system promotes the formation of biofilms^11–13^. Biofilms are communities of bacteria surrounded by an extracellular matrix (ECM) composed largely of proteins and extracellular DNA that not only provides support for the bacterial cells, but also helps to protect them from both immune defenses and antibiotic treatment^14–17^. Biofilm formation is not a trivial concern as the contribution of biofilms to treatment resistant infections, especially in the presence of foreign materials (i.e. mechanical heart valves, prosthetic joints, catheters, etc.), is well established. When prosthetic material is placed in the body, it rapidly accumulates a coating of host proteins such as fibrinogen and fibronectin^18,19^. In the case of surfaces that have been coated with a matrix in this way, bacterial cell wall anchored proteins that specifically recognize various host matrix components are important^14^. These cell wall anchored proteins include the microbial surface components recognizing adhesive matrix molecules (MSCRAMMs) which recognize and bind to numerous host substrates including fibrinogen, collagen and fibronectin^20–22^. MSCRAMMs are negatively regulated by the *agr* system, so it is argued that QSI will downregulate virulence factors but upregulate biofilm (reviewed in^6^). Indeed, many reports have demonstrated the link between mutated *agr* and enhanced biofilm formation in vitro^23–26^. However, while *agr* inhibition may drive *S. aureus* biofilm formation in vitro, dissemination from biofilms requires *agr* activation and *agr* mutants have been demonstrated to have reduced virulence in biofilm models^6,13,25,26^. Additionally, it is not clear that inhibiting *agr* actually promotes biofilm formation in environments that mimic those likely to be encountered by *S. aureus* in a host^6^. Studies utilizing QSIs in mouse models of *S. aureus* skin and soft tissue infections (SSTI) have reported a reduction in CFUs but have not attempted to assess effects on biofilm development, as biofilm formation is not typically considered to be a clinically relevant concern in SSTI models^9,27,28^. Importantly, at this time, almost no in vivo studies have been conducted to assess the impact of QSI treatment on biofilm development with in vivo infection models involving foreign material even though these constitute the situation most likely to be relevant^29^. Consequently, determining the response of *S. aureus* to *agr* inhibition both under various assay conditions in vitro and using in vivo models of biofilm infection is a necessary step in the development of QSIs as a potential therapeutic option.

## Results

### *The role of agr* in *S. aureus* biofilm formation in vitro is assay and strain dependent

**Figure 1:**
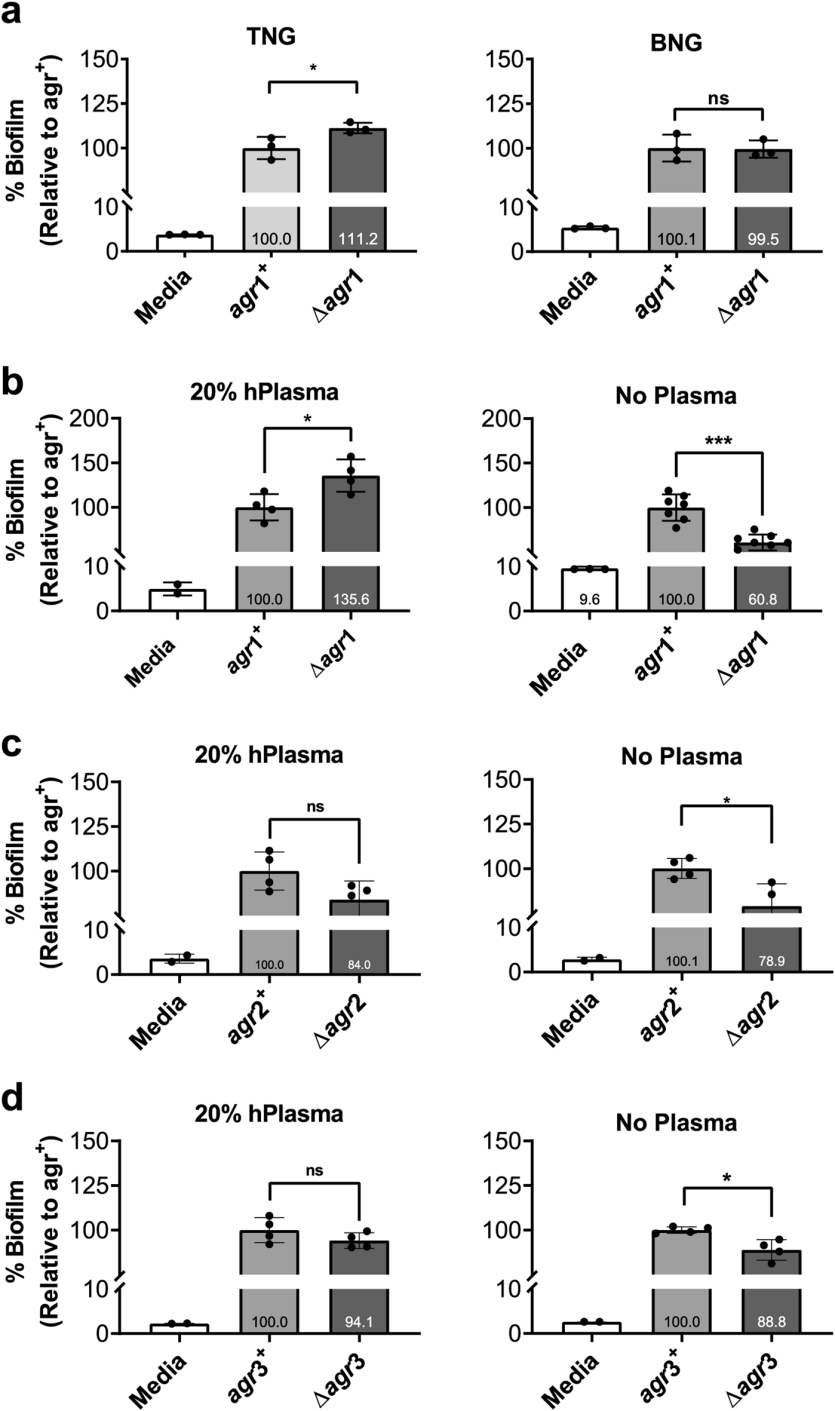
Strain and condition dependence of *Δagr* biofilm enhancement in vitro. (**a**) Comparison of *agr*1 biofilm grown with 20% human plasma in TSB + 3% NaCl + 0.05% Dextrose (TNG) versus BHI + 3% NaCl + 0.05% Dextrose (BNG). (**b**) Comparison of *agr*1 biofilm grown in TNG with 20% human plasma (left) or without (right). (**c**) Comparison of *agr*2 grown in TNG with 20% human plasma (left) or without (right). (**d**) Comparison of *agr*3 grown in TNG with 20% human plasma (left) or without (right). Percent biofilm growth is relative to the *agr* + strain. The *agr*1 strain is AH1263 and its isogenic deletion mutant is AH1292, and the *agr*2 and *agr*3 are the *S. aureus* strains 502A and MW2, respectively with their isogenic deletion mutants. Data represent ≥ 3 biological replicates and are expressed as the mean ± SD (*p < 0.05, ***p < 0.001, ****p < 0.0001, ns = not significant).

Previous in vitro studies have demonstrated that *S. aureus Δagr* strains, which serve as a useful analog for the effects of QSIs, exhibit a greater degree of biofilm formation than *agr* + strains^11–13^. However, we hypothesized that biofilm development by *S. aureus Δagr* strains would vary based on assay conditions and *agr* allele. To address this hypothesis, *S. aureus* biofilms were grown and quantified according to a modified version of the crystal violet assay detailed by Cassat et al.^30^. When grown for 24 h on plates pre-coated in human plasma in tryptic soy broth supplemented with 3% w/v NaCl and 0.5% w/v dextrose (TNG), an *S. aureus agr*1 strain (AH1263 = *agr*1^+^, AH1292 = *Δagr*1) adhered to the established pattern with the *Δagr* mutant producing significantly more biofilm than its *agr* + equivalent (Fig. 1a, left). However, when grown in similarly supplemented brain heart infusion media (BNG), no difference in biofilm production was observed regardless of the presence of *agr* (Fig. 1a, right). The crystal violet assay was developed to analyze robust biofilms in 96-well plates that had been pre-coated with 20% v/v human plasma. We therefore compared the same strains when grown in TNG with or without any plate pre-coating. In this setting, the *agr*1 + strain exhibited increased biofilm production relative to *Δagr*1, in contrast to observations with plasma pre-coating (Fig. 1b, left vs. right). In assessing the impact of the *agr* allele, the *agr*2 (502A = *agr*2^+^, 502A *Δagr* = *Δagr*2) and the *agr*3 (MW2 = *agr3*^+^, MW2 *Δagr* = *Δagr*3) strains did not exhibit a difference in biofilm production relative to the *Δagr* counterparts with plasma pre-coating, however both produced more biofilm relative to their *Δagr* counterparts when grown without plasma pre-coating (Fig. 1c,d)*.* These results suggest that assay conditions (i.e.—media and pre-coating status) and *S. aureus agr* allele play important roles in in vitro biofilm assays. Importantly, comparing raw optical density values and not normalized data between strains can be very informative. While the trends noted above do not change, it becomes very clear that the *agr3* strain is the strongest biofilm former with *agr2* as second, and the *agr1* strain is actually the least strong biofilm former (Supplemental Fig. 1).

### *Staphylococcus aureus Δagr* biofilms are enhanced during biofilm development

**Figure 2:**
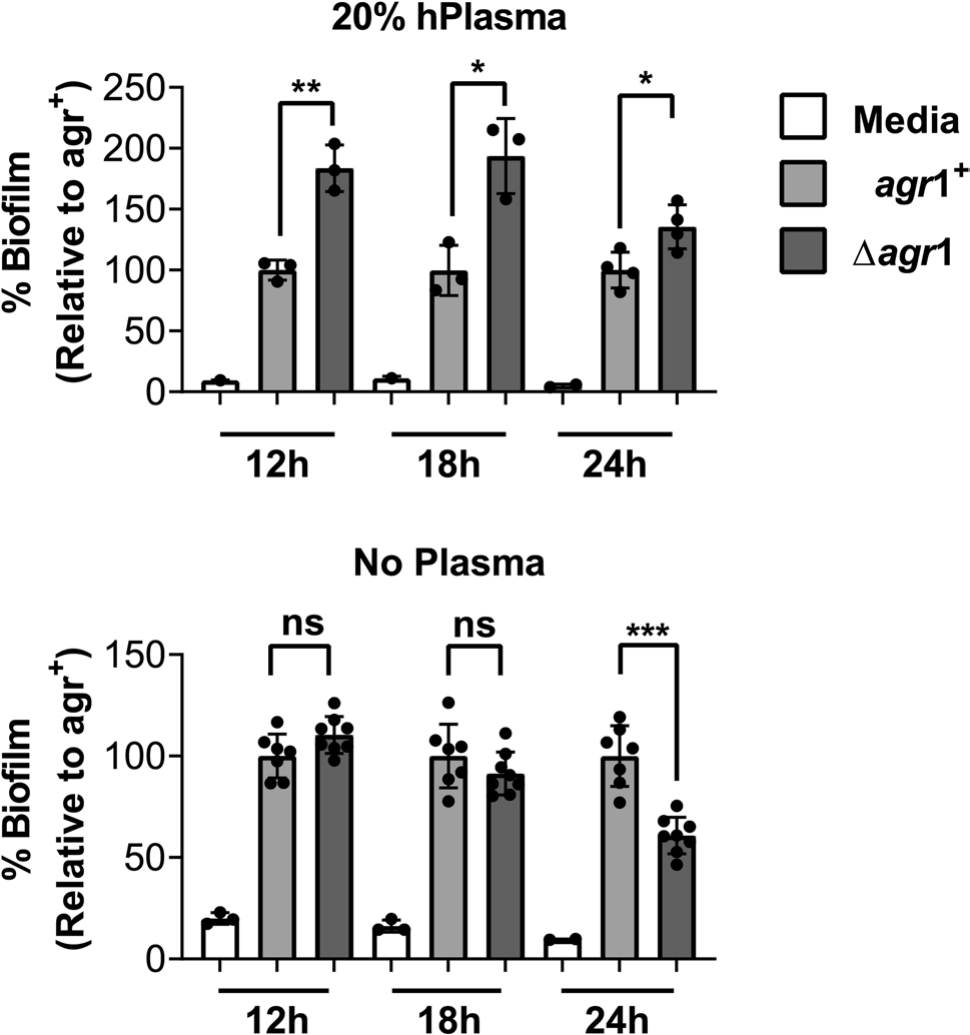
Enhanced S. aureus Δagr1 biofilm formation in vitro is most pronounced as biofilm forms. (**a**) Comparison of biofilm development after 12, 18, and 24 h of growth in TNG with 20% human plasma pre-coating (top) or without plasma pre-coating (bottom). 24 h data is re-graphed from Fig. 1 for comparison. This is *agr*1 strain is AH1263 and its isogenic deletion mutant is AH1292. Data represent ≥ 3 replicates and are expressed as the mean ± SD and data is normalized to the agr + at each respective time point (***p < 0.001, ****p < 0.0001, ns = not significant).

To determine if the trends we had observed above were relevant at different stages of biofilm development, we compared biofilm formation from 12 to 24 h using the *agr*1 and *Δagr*1strains grown in TNG on either pre-coated or uncoated 96-well polystyrene plates. When grown on pre-coated plates, the *Δagr* strain formed a larger biofilm at all time points relative to the *agr* + strain (Fig. 2, top). However, by 24 h the difference between strains had decreased from over 75% at 12 and 18 h to ~ 35% by 24 h. When grown on uncoated plates, no difference in biofilm development was observed between the *agr* + and *Δagr* strains at the 12- and 18-h time points, but by 24 h, the *agr* + strain had produced significantly more biofilm than the *Δagr* strain (Fig. 2, bottom). These data demonstrate that not only do assay conditions greatly impact biofilm formation, but temporal measurements can have important impacts on interpretation of biofilm formation.

### *Staphylococcus aureus* biofilm formation on polystyrene is replicated on a medically relevant plastic

**Figure 3:**
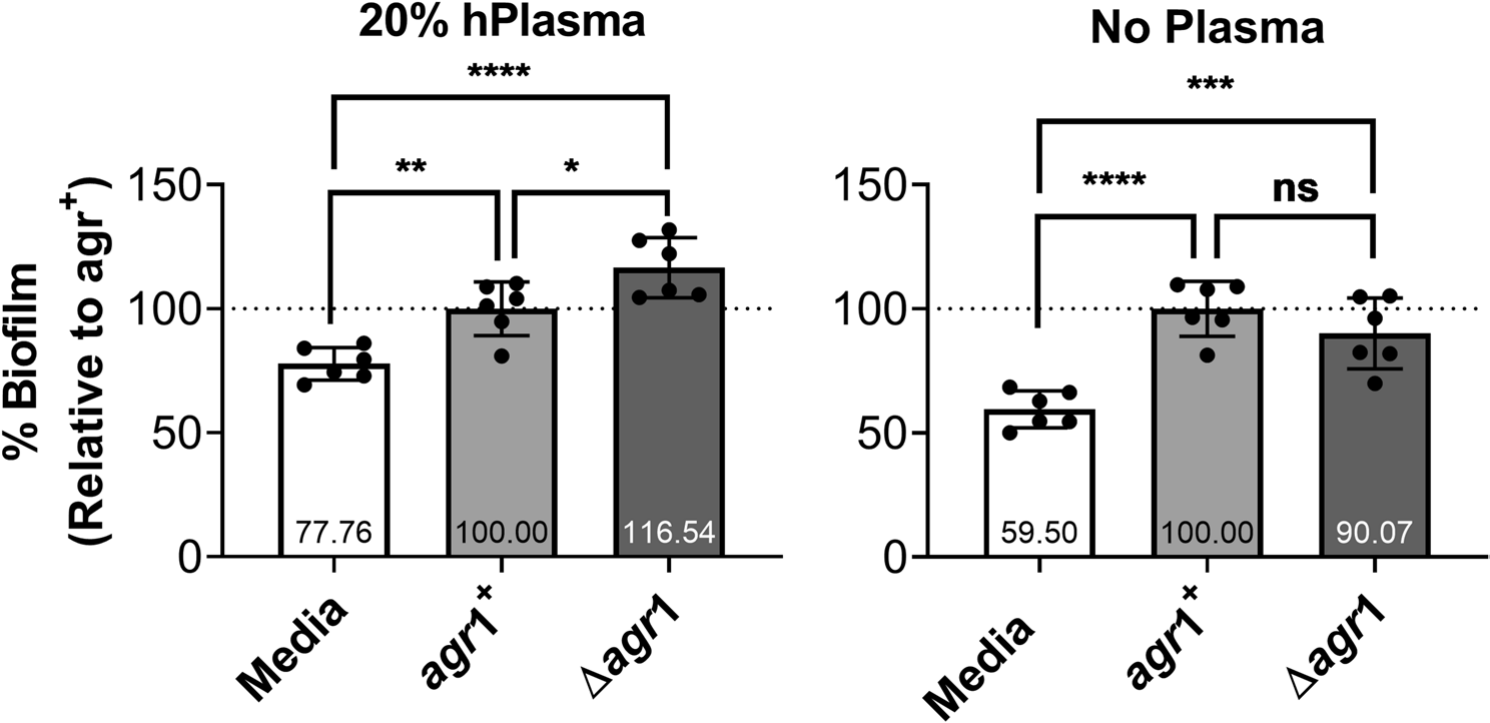
Biofilm enhancement on catheters is also assay dependent. Comparison of biofilm development on 1-cm long segments of BD Vialon catheters with 20% human plasma pre-coating (left) or without plasma pre-coating (right). This is *agr*1 strain is AH1263 and its isogenic deletion mutant is AH1292. Data are three catheter replicates repeated twice and are expressed as the mean ± SD (*p < 0.05, **p < 0.01, ***p < 0.001, ****p < 0.0001, ns = not significant).

The experiments described above, and those generally found in the literature, assess *S. aureus* biofilm formation in polystyrene plates, however, polystyrene is not a clinically relevant material as it is not representative of the materials that are used in human patients^30^. To address the role of *S. aureus agr* in biofilm formation on a material used in human patients, we utilized venous catheter segments and further modified the crystal violet plate assay. Unlike polystyrene plates, the catheters (made from BD Vialon, a proprietary material intended for intravenous insertion) adsorbed the crystal violet stain resulting in a high level of background (Fig. 3). However, catheters incubated with bacteria and then stained with crystal violet demonstrated increased signal above background, indicating that biofilm could be quantified on the catheters. Similar to the plate-based assay, the *S. aureus Δagr*1 isolate produced more biofilm relative to *agr* + when grown with human plasma pre-coating (Fig. 3, left). However, when grown on uncoated catheters, no difference in *S. aureus* biofilm production was observed, although the trend followed that observed in plate-based assays (Fig. 3, right). While polystyrene is not inserted into humans, in these specific conditions it recapitulates a venous catheter well.

### Bacterial burden does not necessarily correlate with amount of biofilm

**Figure 4:**
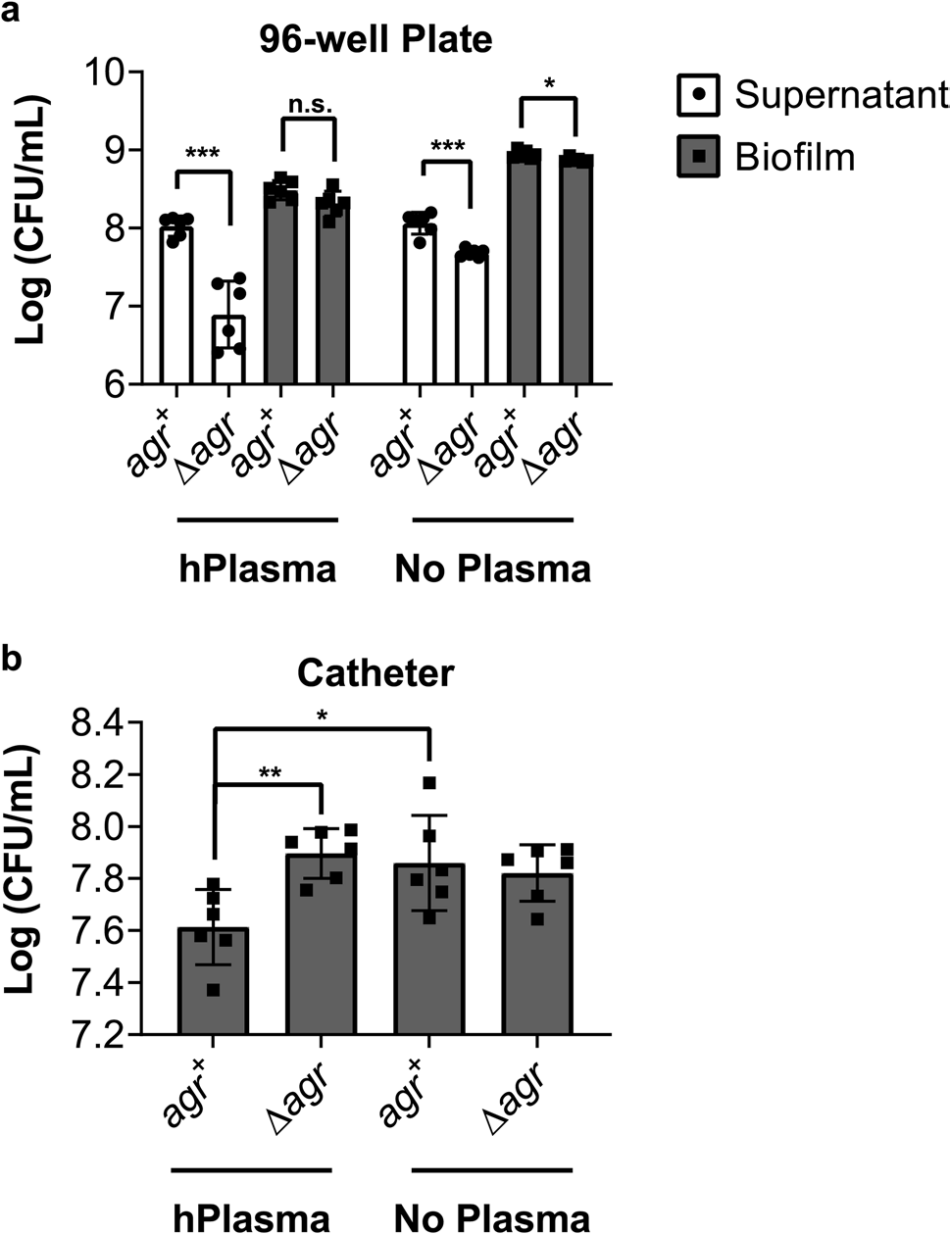
CFUs do not reliably correlate with amount of biofilm. (**a**) Comparison of CFUs in culture supernatant and biofilm-associated when grown in microtiter plates with and without 20% human plasma pre-coating. (**b**) Comparison of CFUs in biofilms grown on 1-cm catheter segments with and without 20% human plasma pre-coating. This is *agr*1 strain is AH1263 and its isogenic deletion mutant is AH1292. Data represent ≥ 3 biological replicates and are expressed as the mean ± SD (*p*-values: *p < 0.05, **p < 0.01, ***p < 0.001, ns = not significant).

Biofilms are composed not only of the bacteria, but also of the ECM in which they are embedded. Therefore, while the crystal violet assay allows for total biofilm quantification, the amount of biofilm measured may or may not be a proxy for the actual number of viable bacteria present. To determine whether or not the amount of biofilm correlates with the total bacterial burden, we repeated both the plate- and catheter-based assays using the *agr1* + and *Δagr1* isolates described above and assessed the number of biofilm-associated colony forming units (CFUs). When grown on plates with plasma pre-coating, the number of CFUs present failed to follow the trend associated with the amount of biofilm measured in the crystal violet assay (Fig. 1b), as there was not a significant *agr*-dependent difference in the number of bacteria associated with the biofilm (Fig. 4a). However, significantly fewer *S. aureus Δagr* CFUs were recovered from the supernatant as compared to the *agr* + strain (Fig. 4a). Although statistically significant, only a minor decrease in biofilm-associated CFUs were observed in microtiter plates without human plasma pre-coating and the reduced CFU content of the *Δagr* supernatant was again noted (Fig. 4a). Observed differences in CFUs for *agr1* + and *Δagr1 S. aureus* biofilms grown on catheter segments correlated with the crystal violet experiments. When grown on catheter segments with plasma pre-coating the *Δagr* biofilm contained more bacteria (CFUs) than the *agr* + biofilm, but when grown on catheter segments without plasma pre-coating, no difference in CFUs was observed between *agr* + and *Δagr* biofilms (Fig. 4b). These experiments demonstrate that bacterial burden does not necessarily correlate with the amount of biofilm present and that the relationship between the amount of biofilm and the number of bacteria present is substrate dependent.

### *Staphylococcus aureus agr* drives morbidity, but not biofilm formation, in a mouse model of subcutaneous implant associated infection

**Figure 5:**
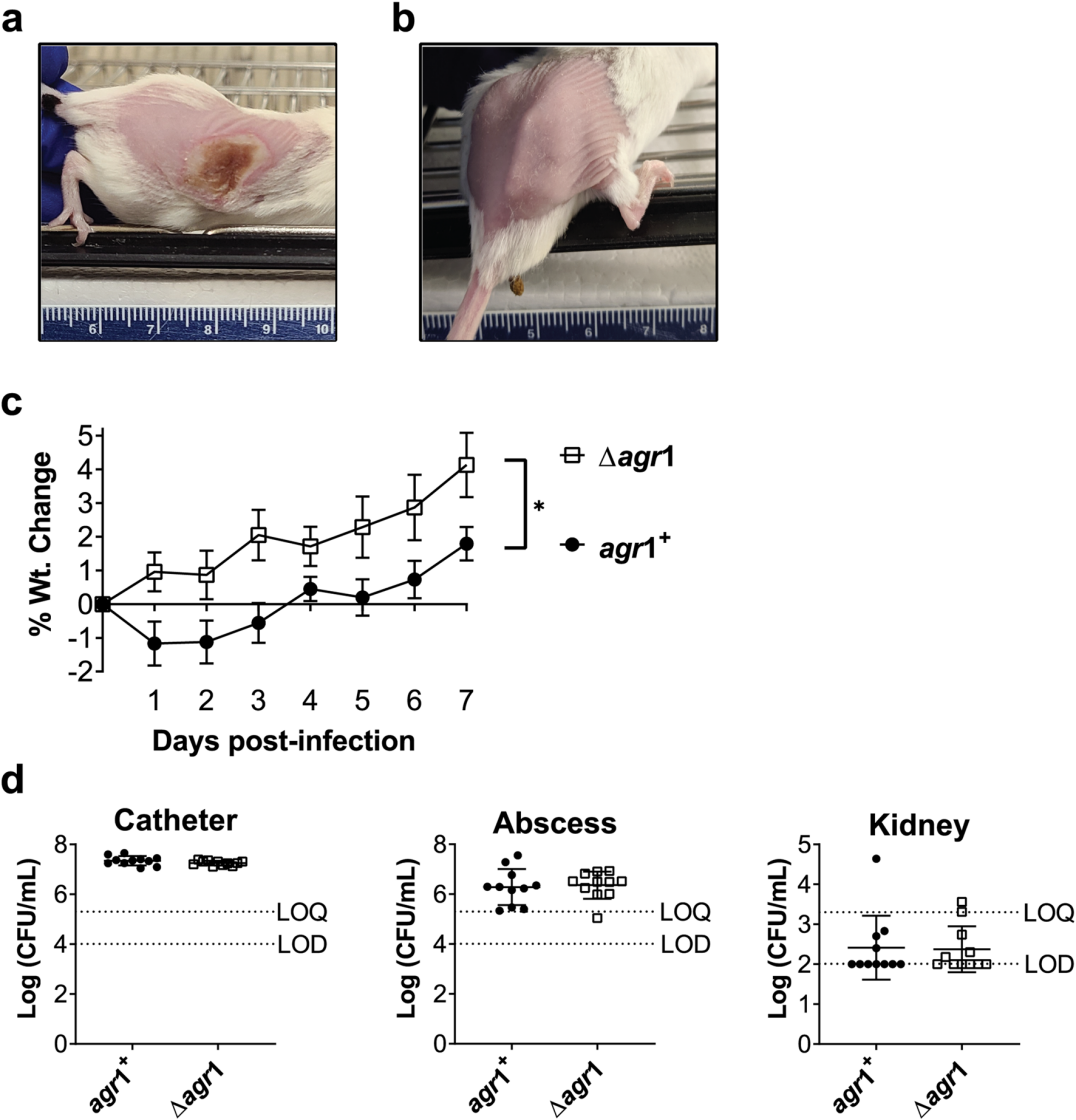
*agr*1^+^ is more pathogenic than Δ*agr*1 in a mouse model of implant associated infection. Gross pathology on day 3 post-infection of a subcutaneous (**a**) compared to an implant-associated (**b**) infection with *agr1* + *S. aureus* in a BALB/c mouse. (**c**) Percent weight change of mice over 7 day infection. (**d**) Bacterial burden (CFUs) on implanted catheter (left), surrounding abscess (middle), and in the kidneys (right) on day 7 post-infection. Data are the average of two experiments with a cumulative total of n = 11 mice per group and are expressed as the mean ± SEM (*p*-values: *p < 0.05).

While in vitro experiments are useful, they are unable to fully replicate the complexity of infection development in a living host with a functional immune response. We utilized a mouse model of implant associated infection in which mice had sterile catheter segments implanted subcutaneously and, after allowing two days for the implanted catheters to become coated with host proteins, catheters were inoculated via the catheter lumen with either *agr1* + or *Δagr1 S. aureus*. Interestingly, while mice given a direct subcutaneous injection of *agr1* + *S. aureus* developed large, necrotic lesions (Fig. 5a), most of the mice injected via the catheter lumen did not develop a visible lesion and in the few that did, the lesion was limited to either the catheter insertion site (posterior) or inoculation site (anterior) (Fig. 5a vs. b).

Mice were weighed daily to assess morbidity over the seven-day experiment. As expected, mice that were infected with the *agr1* + strain initially lost weight before beginning to gain weight again by day three, but mice infected with the *Δagr1* strain continued to gain weight throughout the course of the infection (Fig. 5c). Using weight loss as an indicator of overall morbidity, these data suggest that in this model of biofilm infection, *agr* + *S. aureus* remains more virulent relative to *Δagr*. On the seventh day post-infection, we collected the implanted catheters, the inflamed tissue surrounding the catheters (labeled abscess) and the kidneys to assess the bacterial burden at each of these locations. Importantly, no difference in CFUs was observed at any of the three sites evaluated (Fig. 5d). Additionally, CFUs from the kidney (a blood filtering organ) were at or near the limit of detection (Fig. 5d), this demonstrates that dissemination from the infection site was minimal with either strain. These data were recapitulated with *S. aureus agr2* + and *Δagr2* strains (Supplemental Fig. 2). These in vivo experiments demonstrate that, even in a biofilm infection model, *Δagr* strains are less virulent than their counterpart *agr* + strains and supports the use of QSIs as a viable treatment option for *S. aureus* infections even in the presence of foreign materials.

## Discussion

The development of novel drug therapies to combat infections involving drug resistant organisms including MRSA is crucial to ensure that effective treatments for these infections remain available. Accordingly, QSIs that target the *agr* operon represent a therapeutic option for attenuating *S. aureus* virulence and so enhancing immune clearance of these infections. Here, using *agr* deletion mutants, we have shown that a major criticism of QSIs, that *agr* inhibition will inevitably lead to enhanced biofilm development, is not substantiated under a variety of experimental conditions including, crucially, in an in vivo model of implant associated infection.

In our experiments, we utilized *agr* deletion mutants of *S. aureus* as we expected their behavior to approximate the behavior of *agr* + *S. aureus* in the presence of a QSI. However, as QSIs do not completely block *agr* function their effects on biofilm formation are likely to be less pronounced than those of *agr* deletion^31,32^. Therefore, Δ*agr* strains are anticipated to be a more stringent assessment of the possible QSI phenotype, i.e. lack or inhibition of *agr* function is reported to lead to enhanced biofilm growth^11,13,33^. Other studies have demonstrated that the relationship between biofilm development and *agr* function varies depending on factors including glucose availability and *S. aureus* strain^34–36^. In contrast, our in vitro experiments demonstrate that enhanced biofilm development in the absence of functional quorum sensing depends not only on growth conditions but also on the *agr* allele of *S. aureus* utilized.

Here, microtiter plates and catheter segments were either pre-coated with 20% human plasma or left uncoated to simulate two possible clinical scenarios: (1) placement of sterile implants and catheters , that rapidly acquire a coating of host matrix that ultimately make them much more hospitable for the attachment and growth of microorganisms (by far the most common scenario)^18,19^ and (2) inadvertent contamination of materials/devices prior to implantation. In this second scenario, the contaminating bacteria must become established without the benefit of a pre-formed host matrix to facilitate their attachment. We observed that the absence of *agr* was only associated with increased biofilm under narrow conditions, specifically when the Δ*agr*1 strain was grown in TNG with plasma pre-coating. With all other strains and under all other experimental conditions evaluated, either no difference in biofilm formation was observed between *agr* + and *Δagr S. aureus* or the lack of *agr* functionality was associated with a small decrease in biofilm formed. Isolates of all four *agr* types are implicated in causing disease in humans, so it is important to note that enhanced biofilm formation with Δ*agr* was only observed in the *agr*1 strain. No *agr*4 strain was utilized because in the United States *agr*4 strains are isolated from infected patients much less frequently than strains of the other *agr* types. While only *agr* alleles 1, 2, or 3 were tested here, the lack of *agr* function was only associated with increased biofilm in a limited set of circumstances, which suggest that in vitro assays may not necessarily predict in vivo outcomes.

Time courses of biofilm formation by *S. aureus* obviously demonstrate a greater degree of biofilm development later relative to earlier time points. Importantly, few of these studies have been reported, with one conducted using a human clinical isolate of *S. aureus*, the other utilizing isolates from cases of bovine mastitis^37,38^. However, it appears that no studies to date have specifically investigated the relationship between *agr* status and biofilm growth across multiple time points. Therefore, we conducted time course experiments to elucidate possible time dependence in the relationship between biofilm growth and *agr* status utilizing the *agr*1 strain, the only strain showing an increase in biofilm growth in the absence of *agr*. We found that when grown with pre-coating, the absence of *agr* greatly enhanced biofilm formation compared to the *agr* + strain during earlier timepoints. However, by 24 h the *agr*-dependent difference had decreased. In contrast, there was no *agr*-dependent difference seen on the uncoated plates until 24 h and the *agr* + strain made more biofilm at this time. Although biofilms were not grown beyond the 24-h time point, based on the trends observed we predict that given more time the *agr* + strain would possibly have matched or surpassed the Δ*agr* strain in biofilm production on coated plates. Conversely, based on the analysis of the uncoated plates, we predict that past 24 h the *agr* + strain would have maintained an increased degree of biofilm development relative to the Δ*agr* strain. This is important because time points beyond 24 h post-infection are likely to be most relevant when considering clinical scenarios in which an infection is complicated by biofilm growth. Such infections have a propensity to become chronic, requiring multiple treatment modalities and potentially months of therapy, therefore longer time points are more likely to reflect the degree of biofilm development that would be encountered in patients^39–41^. Future studies should assess biofilm formation at time points beyond 24 h and on a wider variety of medically utilized materials to more closely approximate clinically relevant conditions.

Crystal violet staining of biofilm grown in plasma coated plates detects both human and bacterial ECM. While numerous studies have evaluated in vitro biofilm formation by utilizing various methods to quantify the total amount of biofilm present, relatively few studies have measured biofilm associated CFUs in vitro^11,13,42,43^. Studies that have measured CFUs have not compared these numbers to total biofilm and moreover, we were unable to locate any studies that focused on biofilm associated CFUs in relation to *agr* status. While the ECM helps to facilitate *S. aureus* persistence in biofilm associated infections, it is the bacteria that are ultimately responsible for morbidity and mortality. Increased biofilm production by *Δagr* strains may not be particularly important if the number of bacteria within is the same as in biofilm produced by *agr* + strains. In support of this idea, the results of our CFU measurements in combination with the crystal violet biofilm assays show that amount of biofilm is not necessarily a reliable proxy for the bacterial burden. This relationship appears to vary by substrate material with bacterial burden correlating with total amount of biofilm on catheter segments (a clinically relevant material) but not on polystyrene plates. As a result, it will be important to investigate CFU trends on other medically relevant materials, particularly those used in joint replacements (titanium, cobalt, other plastics, etc.), and on devices inserted in regions of blood flow, such as venous catheters, as these surfaces have a propensity to support biofilm.

While in vitro observations are undeniably valuable, conditions in these experiments do not completely replicate those encountered by *S. aureus* in vivo. When QSIs are used in vivo, in the context of SSTIs, QSI treatment results in an infection course that is less severe than infections caused by untreated *agr* + *S. aureus* and that resembles infections caused by Δ*agr* strains^9,27,28^. However, these studies did not attempt to evaluate biofilm formation, nor are they representative of the clinical setting in which biofilms are likely to be of greatest concern, that is, in the presence of foreign material. In vivo models of device associated *S. aureus* infection that are designed to allow for biofilm quantification have been validated for various materials and infection types including prosthetic joint infections and catheter associated infections^30,44,45^. Despite this, the effects of *agr* function on biofilm development in vivo remain largely unexplored. One study using the QSI FS8 in a rat model of implant associated infection with an *agr*III *S. aureus* found that FS8 treatment resulted in a decrease in implant associated CFUs relative to untreated controls while the combination of FS8 and tigecycline led to an even greater CFU reduction^46,47^. FS8 is a derivative of RNAIII inhibiting peptide that reportedly inhibits quorum sensing by blocking the action of RNAIII at the P3 promoter of the *agr* operon^46^. In a murine model of implant associated infection in which mice were infected with an *agr*I strain of *S. aureus*, we observed no difference in bacterial burden between the *agr* + and the Δ*agr* strain at any site measured including the implanted catheter itself, the abscess tissue immediately surrounding the catheter or the kidneys. In contrast to the large, necrotic lesions observed in murine SSTI models, we observed only small, relatively superficial lesions in a small number of mice utilized in our model of implant associated infection. Our CFU measurements illustrate that the number of bacteria associated with the implanted catheter are far greater than those that successfully disseminated into the surrounding tissue or organs, independent of *S. aureus agr* status. The lack of visible lesions on most mice can likely be explained by the fact that most of the infecting bacteria remained inside of and associated with the catheter rather than disseminating and causing obvious pathology in the surrounding tissue. Mice infected with *agr* + *S. aureus* exhibited greater morbidity than those infected with Δ*agr S. aureus* as evidenced by the difference in percent weight change over the course of infection. Mice infected with *agr* + *S. aureus* initially lost weight before beginning to gain again while mice infected with Δ*agr S. aureus* never lost weight and continued to grow throughout the course of infection. Importantly, these divergent growth trajectories show that even in a biofilm infection *S. aureus* with functional *agr* causes a more virulent infection with greater physiological cost to the host. Collectively, these results show that the absence of *agr* function is associated with reduced overall morbidity based on weight loss without a concurrent increase in biofilm associated CFUs (i.e. CFUs on catheter segments). The total absence of *agr,* as in Δ*agr* strains of *S. aureus,* is not a perfect analog for the effects of QSIs. However, these results suggest that the concern of QSI therapy leading to enhanced biofilm development may be of limited relevance in vivo. Importantly, QSI therapy non-competitively and reversibly suppresses *agr* function so any biofilm enhancement occurring in the presence of a QSI would be expected to be even less pronounced than what is observed with a Δ*agr* strain. Future studies are planned to assess biofilm development in implant associated infections in the context of QSI therapy. Since Δ*agr S. aureus* is a useful, but not perfect stand-in for the effects of QSIs, these studies will be important in order to confirm that the lack of biofilm enhancement observed with Δ*agr* strains as shown here holds true when *agr* is functional but inhibited. Furthermore, other Staphylococci are also medically relevant biofilm formers and also possess *agr* systems^3,5,6^. It will therefore be essential to assess the effects of QSI and *agr* functionality in the wider Staphylococci (e.g.—*S. epidermidis*).

Finally, there is also evidence that the combination of antibiotics and QSIs have an additive therapeutic effect in both SSTIs and biofilm associated infections^46,48,49^. Antibiotic therapy is the central component of the standard of care for all types of bacterial infections in human patients. The use of QSIs as an adjunct to antibiotic therapy would more closely approximate the setting in which QSIs are most likely to be clinically useful and would generate valuable information about their effects on both biofilm development and therapeutic outcome in this setting. Therefore, in future studies it will also be important and informative to utilize a QSI in combination with appropriate antibiotics in models of implant associated infection and SSTI.

## Methods

### Bacterial strains and growth conditions

The *S. aureus* strains utilized were as follows: AH1263 and its *agr* deletion mutant AH1292 (*agr*1), and MW2 and its *agr* deletion mutant (*agr3*) were generously provided by Dr. Alexander Horswill (University of Colorado Anschutz Medical Campus, Aurora, CO). 502A and its *agr* deletion mutant (*agr2*) were provided by the Network on Antimicrobial Resistance in *S. aureus* (NARSA) for distribution by BEI Resources, NIAID, NIH (*S. aureus,* NR-45946 and NR-45957 respectively). No *agr* IV strain was utilized as discussed above. Unless otherwise noted, bacteria were cultured at 37 °C with shaking at 220 rpm in TNG [trypticase soy broth (Becton, Dickinson and Company, Sparks, MD) with 0.5% w/v dextrose (VWR Analytical, Radnor, PA) and 3% w/v NaCl (Fisher Scientific, Waltham, MA)].

### Crystal violet plate assay

*Staphylococcus aureus* biofilms were grown in flat bottomed, polystyrene, 96-well microtiter plates (Corning #3370, Corning, NY) and quantified following a modified version of a protocol previously published by Cassat et al.^30^. Specifically, for assays performed with and without plate pre-coating respectively, 200 μL of either 20% v/v human plasma (Mediatech, Inc., Manassas, VA) or 1X phosphate buffered saline (PBS) was added to plates and incubated overnight at 4 °C. Simultaneously, overnight cultures in duplicate were started by inoculating 3 mL of respective media with either an *agr* + strain of *S. aureus* (e.g. AH1263) or the corresponding *agr* deletion mutant (e.g. AH1292). Following overnight growth, cultures were matched by optical density (OD_600nm_) + /−0.05. OD-matched cultures were then diluted 1:200 ratio in fresh media and placed on ice. The 20% v/v plasma or PBS in the biofilm plate was removed via gentle aspiration and the wells were inoculated with 200 μL of the previously diluted cultures. Plates were set up so that 21 wells were devoted to each biological replicate with sterile TNG as a control, and repeated at least once. Once inoculated, plates were covered with a gas permeable membrane (Sigma Aldrich, St. Louis, MO) and incubated at 37 °C without shaking for 24 h. After incubation, cultures were gently aspirated out of each well, to avoid disturbing any material adhering to the sides or bottom of the wells. From this point, the plates were processed slightly differently depending on whether they had been pre-coated with plasma or not. Wells of plates that had been pre-coated with plasma were washed twice with 200 μL of sterile PBS and then fixed with 200 μL of 100% v/v ethanol. The ethanol was aspirated, and the plate was allowed to dry for 10 min. The biofilm was stained via the addition of 200 μL of 0.1% w/v crystal violet (Sigma Aldrich) to each well and incubated for two minutes before being aspirated. The plate was gently washed twice more with 200 μL of PBS before 100 μL of 100% v/v ethanol was added to each well and the plate was placed on a titer shaker for 10 min to elute the crystal violet. The eluted stain in each well was diluted 1:10 in 100% v/v ethanol in a new plate to be within the linear range of the spectrophotometer. To quantify the amount of biofilm present, absorbance was read at OD_595nm_ nm using a Molecular Devices SpectraMAX 340 spectrophotometer. Plates that were left uncoated were processed using the protocol above with additional modifications to prevent the biofilms from being washed away. At each step that required PBS washes, these plates were washed only once and after each addition of liquid they were centrifuged at 3000×*g* for 2 min. Finally, the 1:10 dilution prior to absorbance readings was not necessary for uncoated plates as the biofilms were not as robust. The protocol described above for plasma coated plates was also performed using the *agr*1 strain with brain heart infusion media (Becton, Dickinson and Company, Sparks, MD) containing 0.5% w/v dextrose (VWR Analytical, Radnor, PA) and 3% w/v NaCl (Fisher Scientific, Waltham, MA) as the growth media.

### Crystal violet catheter assay

18-gauge BD Vialon catheters (BD and Co.) were cut into 1-cm long segments. These catheters are intended for venous insertion when used in human patients. Catheter segments were incubated with 200 μL of either 20% v/v human plasma or PBS overnight at 4 °C. *Staphylococcus aureus* cultures were grown, OD matched, and diluted as described above. Following overnight incubation, the plasma or PBS was removed from each tube via gentle aspiration and 200 μL of the prepared cultures was added and incubated at 37 °C without shaking for 48 h. After incubation, catheter segments (with associated biofilms) were transferred to tubes containing 200 μL of 100% v/v ethanol. The ethanol was aspirated off immediately while taking care not to touch the catheter segments, and then allowed to dry for 10 min. After drying, catheters were stained with 200 μL of 0.1% w/v crystal violet for 2 min before the crystal violet was removed. Catheters were then washed twice with 1 mL of PBS. Importantly, during the first wash, 100 μL of PBS was gently pipetted through the catheter lumen to ensure that unbound crystal violet was removed. After washing, 200 μL of 100% v/v ethanol was added to each tube and the tubes were shaken for 10 min to elute the stain. Tubes were briefly vortexed before having 100 μL of their contents transferred to a 96-well plate for OD_595nm_ measurement. As noted in the figure legends, each experiment was performed with multiple catheter segments and was repeated at least once.

### Colony forming unit quantification

96-well microtiter plates containing biofilms were prepared as described above. After the 24-h incubation, the culture supernatant from each well was gently harvested. Biofilms were then washed with 200 μL of PBS with 0.1% Triton X-100 and repeatedly pipetted to disperse biofilm. This low amount of surfactant was verified to have no effect on bacterial viability (data not shown). The dispersed biofilm and harvested supernatant were each serially diluted in PBS and plated on trypticase soy agar containing 5% sheep’s blood (BD and Co.) for incubation, 24 h at 37 °C. After incubation, colonies on each plate were counted to allow CFU quantification. Dilutions containing 20–200 colonies were used unless near the limit of quantitation. Catheter segments were incubated as described above and then transferred to tubes containing 1 mL of PBS with 0.1% Triton X-100 for dispersion by sonication. Dispersed bacteria were plated and enumerated as described.

### Mouse model of implant associated infection

Animal studies described herein were approved by the Institutional Animal Care and Use Committee (IACUC) of the University of New Mexico Health Sciences Center (Animal Welfare Assurance number 19-200,873-HSC) and conducted in accordance to recommendations in the Guide for the Care and Use of Laboratory Animals^50^, ARRIVE^51^ (indicated by lowercase Roman numerals), the Animal Welfare Act, and U.S. federal law.

#### Methods

Eight- to 12-week-old, age-matched female BALB/c mice were shaved and chemically depilated on day −3 (3 days prior to infection) and on day −2, a 1-cm segment of sterile 18-gauge BD Vialon catheter was inserted subcutaneously from the posterior direction into the right flank. On day 0, a 5 μL suspension of 2 × 10^7^ CFU of *S. aureus* either *agr* + (control) or Δ*agr* was injected into the catheter lumen from the anterior direction. Mice were assessed and weighed daily for 7 days and then catheter segments, the skin surrounding the catheters and the kidneys were collected on day 7. Tissue samples were mechanically homogenized, serially diluted in PBS, and then plated on sheep blood agar to determine bacterial burden. Catheters were harvested in 1 mL of PBS. The lumen was flushed with a pipette and the catheter sonicated to disperse bacteria; the resulting suspensions were serially plated as the tissue samples above.

#### ARRIVE statements

In each study we were comparing *S. aureus agr* + (control) to the isogenic Δ*agr* strain (i-a). The experimental unit was one cage of up to six mice, housed based on infecting strain (i-b). The exact number of animals is described in each figure legend, but was between 5–6 animals per experiment, repeated twice for a total of 10–11 animals based on power required in our in vivo dermonecrosis model (Fig. 5a) to detect differences (ii-a,b). Chemical depilation utilizes very low pH solutions, so any mice displaying skin irritation prior to infection were not included and not counted in total n (iii-a,c). For Supplementary Fig. 2, one cage had a water dispenser leak, flooding the cage. Post hoc analysis demonstrated that the weight loss data for this cage were not consistent with the repeat experiment while CFU data was consistent. Therefore, weight loss data were not presented in Supplementary Fig. 2 but the experiment was not repeated in-line with reduce, reuse, and recycle guidelines (iii-b). Mice were grouped by cage based on infectious strain and the technician was aware of the treatment groups at all times, but data was not analyzed until after mouse sacrifice, so they were unaware of results (iv-a,b; v). Outcome measures were cumulative weight-loss for morbidity and tissue burden for clearance (vi). Percent weight change was normalized by calculating the area under the curve (AUC) for each mouse. Weight change AUC and the CFU data was analyzed using a two-tailed Student’s t-test because data was determined normal by Anderson–Darling test; all analysis was completed with GraphPad Prism v9.0.2 (vii). Eight- to 12-week-old, age-matched female specific pathogen-free BALB/c mice were utilized for all experiments (viii). In vivo methods are described above (ix) and data summary and variability are described in the figure legends (x).

### Statistical analyses

Statistical analyses were performed using GraphPad Prism version 9.0.2. The crystal violet microtiter plate and catheter biofilm data were analyzed using a nested t-test (mixed effects model) in which bacterial strains and biological replicates were input as fixed and random effects respectively. The time series data and both the in vitro and in vivo CFU data were analyzed using two-tailed Student’s *t* tests. The results were considered significantly different at a *p* value of < 0.05.

## Supplementary Information

Supplementary Information.
