# Supplementary material for: Nonconformity of biofilm formation in vivo and in vitro based on Staphylococcus aureus accessory gene regulator status
Source: Sci Rep. 2022 Jan 24;12:1251. doi: 10.1038/s41598-022-05382-w (PMC8786897; doi:10.1038/s41598-022-05382-w)
Supplement: Supplementary file 1 — Supplementary Information. [file 41598_2022_5382_MOESM1_ESM.pptx]

## Slide 1
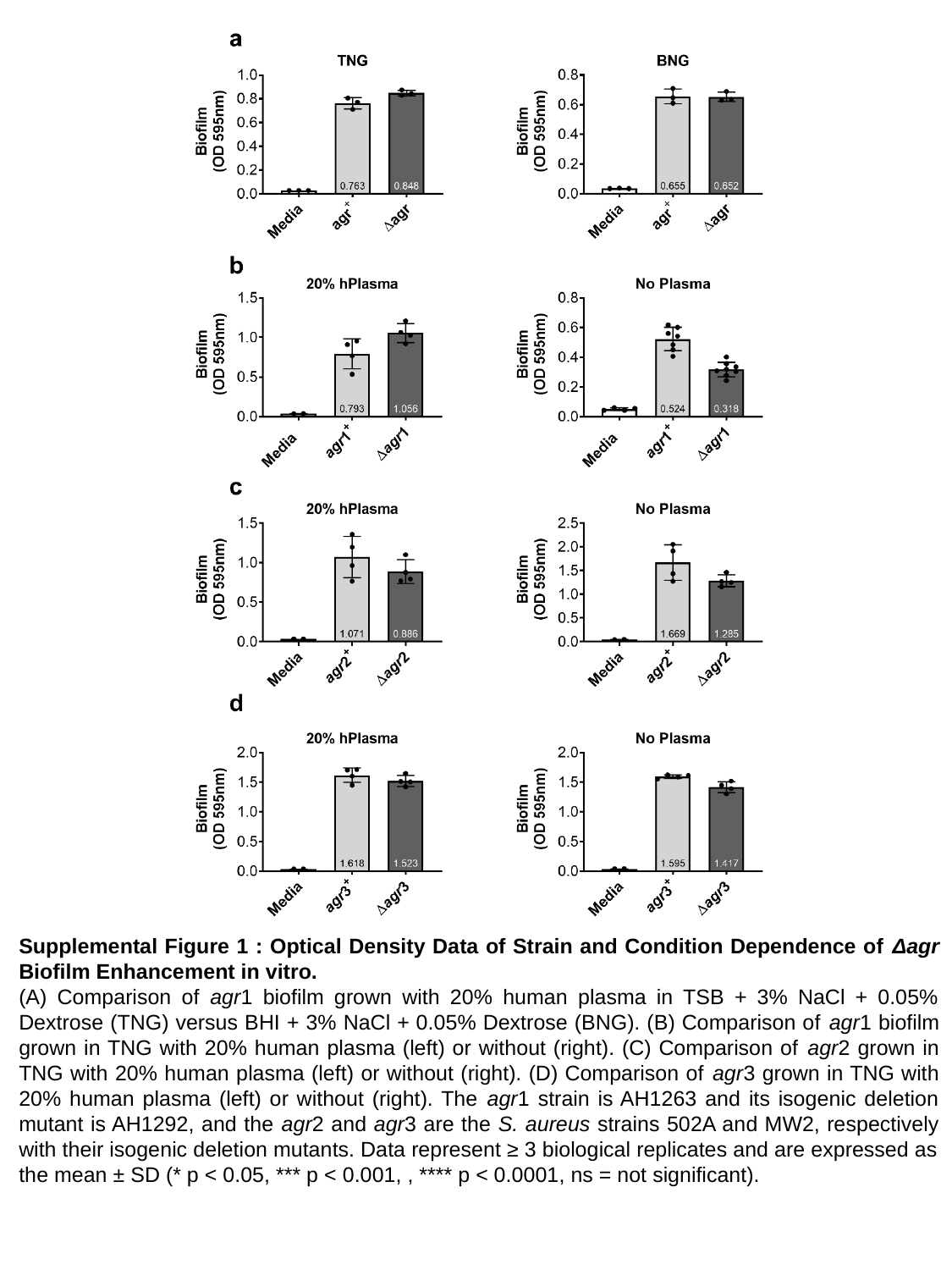

Supplemental Figure 1 : Optical Density Data of Strain and Condition Dependence of Δagr Biofilm Enhancement in vitro.
(A) Comparison of agr1 biofilm grown with 20% human plasma in TSB + 3% NaCl + 0.05% Dextrose (TNG) versus BHI + 3% NaCl + 0.05% Dextrose (BNG). (B) Comparison of agr1 biofilm grown in TNG with 20% human plasma (left) or without (right). (C) Comparison of agr2 grown in TNG with 20% human plasma (left) or without (right). (D) Comparison of agr3 grown in TNG with 20% human plasma (left) or without (right). The agr1 strain is AH1263 and its isogenic deletion mutant is AH1292, and the agr2 and agr3 are the S. aureus strains 502A and MW2, respectively with their isogenic deletion mutants. Data represent ≥ 3 biological replicates and are expressed as the mean ± SD (* p < 0.05, *** p < 0.001, , **** p < 0.0001, ns = not significant).

## Slide 2
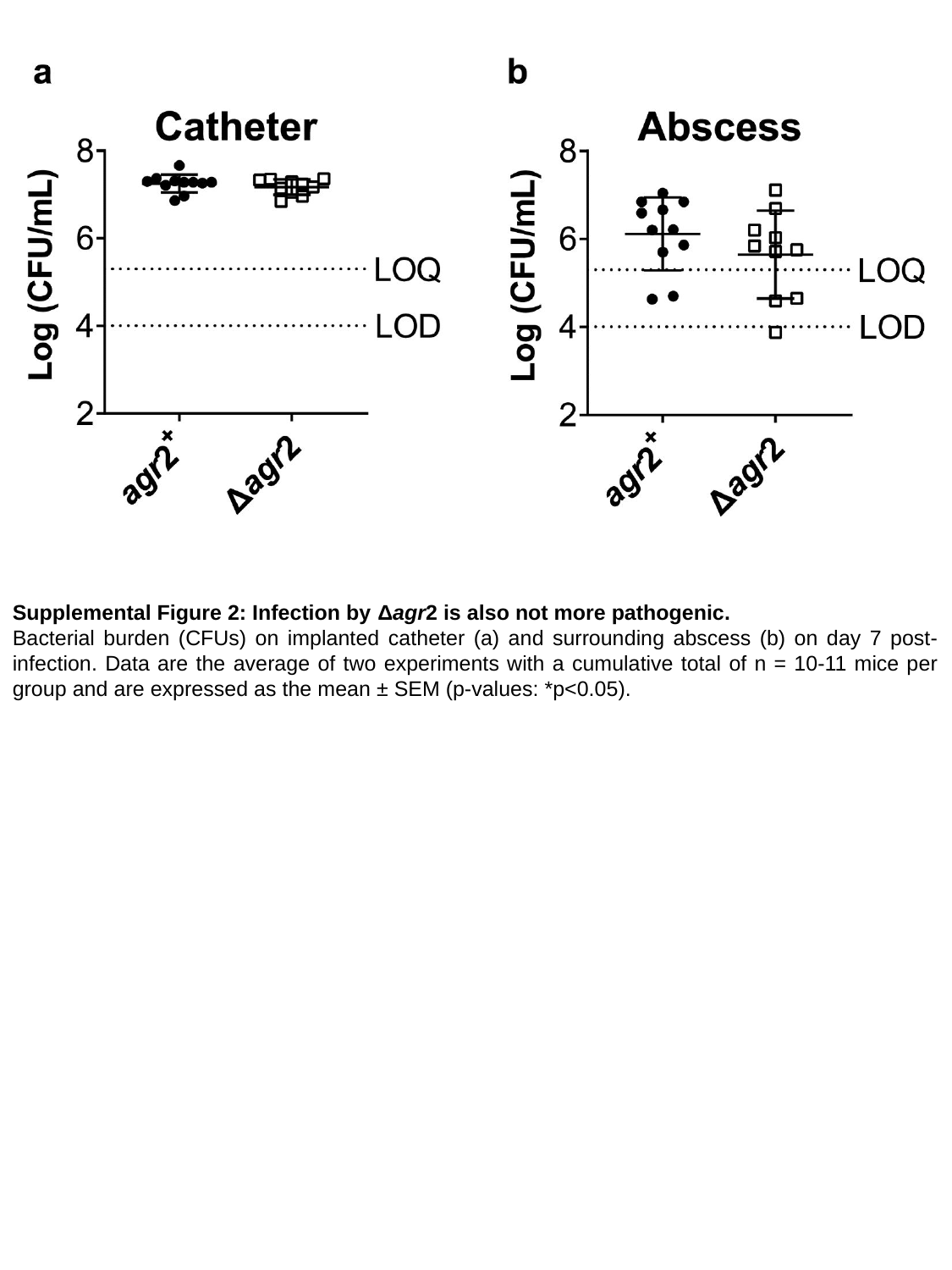

Supplemental Figure 2: Infection by Δagr2 is also not more pathogenic.
Bacterial burden (CFUs) on implanted catheter (a) and surrounding abscess (b) on day 7 post-infection. Data are the average of two experiments with a cumulative total of n = 10-11 mice per group and are expressed as the mean ± SEM (p-values: *p<0.05).
